# Phenotypic and Genetic Characterization and Production Abilities of *Lacticaseibacillus rhamnosus* Strain 484—A New Probiotic Strain Isolated From Human Breast Milk

**DOI:** 10.1002/fsn3.70980

**Published:** 2025-09-26

**Authors:** Natalia Czarnecka, Magdalena Jankowska, Sylwia Nawrot, Katarzyna Nogal‐Nowak, Sławomir Wąsik, Grzegorz Czerwonka

**Affiliations:** ^1^ Division of Microbiology, Institute of Biology Jan Kochanowski University Kielce Poland; ^2^ Regeneris Sp. z o.o. Podzamcze 45 Poland; ^3^ Institute of Physics Jan Kochanowski University Kielce Poland

**Keywords:** antibiotic resistance genes, cell surface hydrophobicity, comparative genomics, human breast milk, *Lacticaseibacillus rhamnosus*, pathogen inhibition, phenotypic characteristics, probiotic product

## Abstract

Recent studies suggest that human breast milk (HBM) is a promising source of probiotic bacteria with potential applications in both medicine and the food industry. Probiotic bacteria, particularly species of the genus *Lactobacillus*, are classified as lactic acid bacteria (LAB). However, probiotic properties are strain‐specific, as not all *Lactobacillus* strains exhibit health benefits or inhibit pathogens. This study evaluated the probiotic potential of a newly isolated strain, *Lacticaseibacillus rhamnosus* strain 484, derived from human milk. Phenotypic and genomic analyses were performed, with *L. rhamnosus* 1.0320 serving as a reference genome. We focused on strain safety for human use and potential health benefits. Strain 484 underwent probiotic characterization and demonstrated strong auto‐ and co‐aggregation abilities, contributing to effective pathogenic bacteria inhibition. The strain also showed bile tolerance, antibiotic sensitivity, and lacked hemolytic and catalase activity, indicating safety and suitability profiles for oral administration. Its resistance to low pH and bile salts indicated survival during gastrointestinal transit and intestinal colonization. Notably, cell surface hydrophobicity (CSH) exceeded that of the well‐known *L. rhamnosus* GG strain, potentially enhancing adhesion to intestinal epithelial cells. Genomic analysis confirmed no antibiotic resistance genes (ARGs) and plasmids, suggesting genetic stability. Overall, *L. rhamnosus* 484 appears to be a safe and promising probiotic candidate with potential applications in both medical and food‐related fields, particularly for oral use in preventing and controlling common pathogens.

## Graphical abstract

Human breast milk (HBM) is a recognized source of probiotic bacteria, with species such as *Lacticaseibacillus rhamnosus* demonstrating considerable potential for applications in both the medical and food industries. In this study, the probiotic potential of strain 484, isolated from HBM, was evaluated through comprehensive phenotypic and genetic analyses. The results confirmed its beneficial properties, including antibacterial activity, bile salt tolerance, and high cell surface hydrophobicity. Moreover, genomic analysis showed that strain 484 is genetically stable and free of antibiotic resistance genes, supporting its suitability as a safe and effective probiotic candidate for medical and industrial use.

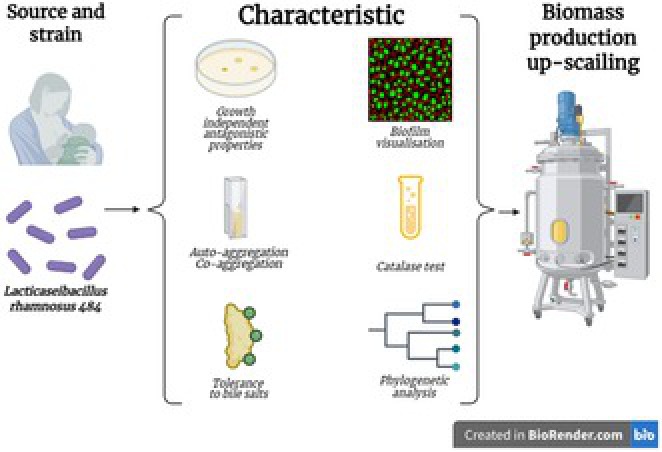

## 1 Introduction

Lactic acid bacteria (LAB) are a diverse group of Gram‐positive microorganisms with beneficial properties, with *Lacticaseibacillus rhamnosus* frequently used in dietary supplements and probiotics (Kim et al. 2020; Zheng et al. 2020). The probiotic potential of LAB strains is determined by their ability to tolerate harsh gastrointestinal conditions, including bile salts and low pH, and by their capacity for auto‐ and co‐aggregation, which facilitates pathogen exclusion (Halloran and Underwood 2019). Safety considerations, such as the absence of hemolytic and catalase activities, are critical for probiotic classification, as these traits reduce the risk of adverse effects (Padmavathi et al. 2018; Spyropoulos et al. 2010). Additionally, probiotic strains can effectively adhere to host cells to facilitate colonization and competitive pathogen inhibition, processes supported by hydrophobicity and biofilm‐forming capabilities (Plaza‐Diaz et al. 2019).

Current research indicates that probiotics derived from human sources, particularly human breast milk (HBM), have unique advantages over those isolated from traditional sources, such as dairy products, pickles, or meat (Fontana et al. 2013). HBM is rich in bioactive compounds—including immunoglobulins, antimicrobial peptides, and beneficial bacteria—that contribute to neonatal immune system development and protect against infections (Fernández et al. 2013). Recent studies have reported that lactobacilli from HBM enhance both innate and adaptive immune responses by activating natural killer cells, stimulating T cells, and expanding regulatory T cell populations (Fontana et al. 2013). Despite these promising findings, a research gap remains in characterizing newly isolated LAB strains from HBM, particularly with respect to safety profiles, adhesion capabilities, and antagonistic activities against pathogens.

In this study, we hypothesized that the newly isolated strain *L. rhamnosus* 484, derived from HBM, exhibits unique phenotypic and genotypic characteristics that make it a promising, safe, and effective probiotic candidate for food and therapeutic applications. Therefore, we evaluated its probiotic potential, including antibacterial activity, safety profile, and adhesion properties, in comparison with reference strains. By identifying unique phenotypic and genotypic traits, we highlight a novel candidate probiotic with potential applications in food products and therapeutic formulations.

## 2 Materials and Methods

### 2.1 Bacterial Strains and Culture Conditions

*L. rhamnosus* strain 484 was identified from a previous research project: “*Development of an innovative line of cosmetics using probiotic bacteria isolated from human milk and Arthrospira platentis (spirulina) biomass*” (NCBiR POIR.01.01.01‐00‐0632/19) implemented by the National Centre for Research and Development, and co‐financed from European Union funds. The strain was isolated from HBM from healthy women (Świętokrzyskie Voivodeship, Poland). Strain 484 was deposited in the Polish Collection of Microorganisms (PCM, www.pcm.org.pl) and assigned the accession number B/00379. The strain was also classified as a lactic acid Gram‐positive bacteria (LAB) of the *L. rhamnosus* species. For optimal growth, the strain was cultivated on de Man, Rogosa, and Sharpe (MRS, Biomaxima) agar plates or MRS broth (Biomaxima), and anaerobically incubated at 37°C in 5% CO_2_ for 24–48 h. Strain 484 was banked in the Microbank Storage Box (Biomaxima) at −80°C.

### 2.2 Auto‐Aggregation

This method was performed as previously described (Reuben et al. 2019), with some modifications. Strain 484 was cultured in MRS broth (Biomaxima) for 24 h, collected, washed twice in phosphate‐buffered saline (PBS; Sigma‐Aldrich), resuspended, and adjusted to an optical density of 0.5 at 600 nm (Jasco V‐660 spectrophotometer). The cell suspension was vortexed for 10 s (Lab Dancer, IKA) and incubated at 37°C for 5 h. Subsequently, the absorbance was measured, and the auto‐aggregation percentage was calculated using the formula:

$$\text{Auto}-\text{aggregation}\;\left(\%\right)=\left[1-A_{5}/A_{0}\right]\times 100$$

where A_5_ represents the optical density (OD) of the microbial suspension (OD = 600 nm) at 5 h, A_0_ represents the optical density of the microbial suspension (OD = 600 nm) at 0 h.

Additionally, auto‐aggregates (clumps) were visualized by cultivating strain 484 for 24 h in MRS medium in a BiostatC bioreactor (Sartorius) at 37°C, and then drying in a spray drier (S‐300, Buchi) in 10% maltodextrin (carrier agent). The pellet was resuspended in sterile saline and subjected to confocal microscopy (VR‐3000 Series, Keyence).

### 2.3 Co‐Aggregation

This method was performed as described (Reuben et al. 2019) with some modifications. Strain 484 was cultured for 24 h in MRS broth at 37°C. *Escherichia coli* PCM 176 was cultured in Lysogeny Broth (LB, Biocorp) for 24 h at 37°C. Then, strain 484 and pathogenic bacterium suspensions (both volumes = 5 mL) were mixed and incubated for 5 h without shaking. Controls were prepared using 10 mL suspensions of each strain only. After incubation, the absorbance (*λ* = 600 nm) of mixtures and controls was measured (Jasco V‐660 spectrophotometer). The co‐aggregation percentage was determined using the following formula:

$$\mathrm{Co}-\text{aggregation}\;\left(\%\right)=\left[\left[\left(1-A_{\mathrm{mix}}\right)/\left(A_{p}+A_{e}\right)\right]/2\right]\times 100$$

where A_p_ represents the OD of the strain 484 cell suspension (OD = 600 nm), A_e_ represents the OD of the *E. coli* pathogen suspension (OD = 600 nm), A_mix_ represents the OD of the bacterial suspension mixture (strain 484 + *E. coli* ) after a 5 h incubation.

### 2.4 Bile Salt Tolerance and Low pH Resistance

Bile salt tolerance test was performed as described by Javed and Chan (Chen et al. 2020; Javed et al. 2022) with some modifications. Strain tolerance to bile salts was examined by preparing four separate tubes containing different bile acid concentrations (0.1%, 0.3%, 0.5%, and 1%) (Pol‐Aura) in MRS broth. To characterize bacterial growth, cultures were grown for 24 h at 37°C, after which pour‐plate inoculations were made on MRS agar plus bile salts at various concentrations. The resistance to low pH test was performed as described by Javed and Chan (Chen et al. 2020; Javed et al. 2022) with some modifications. Growth at a given pH was determined by incubating strain 484 in MRS broth at different pH values (2.0, 3.0, and 4.0). The broth cultures were incubated under anaerobic conditions for 24 h at 37°C, after which 1 mL of culture was inoculated into cooled to 45°C MRS agar, poured on a 90 mm Petri dish, mixed, and left to solidify. The bacterial growth was recorded after 48 h incubation at 37°C. The control was a *L. rhamnosus* 484 culture in MRS broth at standard pH 6.5 (optimal growth conditions).

### 2.5 Hemolytic Activity

Blood hemolysis was assessed by examining hemolytic activity on Columbia agar plus 5% sheep blood (Becton Dickinson) as described (Foongsawat et al. 2023). Strain 484 was inoculated onto agar plates in triplicate. After incubation at 37°C for 48 h, plates were observed for hemolytic reactions. The positive control was *Staphylococcus aureus* (PCM 2054) a reference strain used for hemolysis testing in CAMP tests and showing beta hemolysis (Zhou et al. 2023).

### 2.6 Catalase Tests

Catalase tests were performed using two methods. The first involved the 48‐h culture of strain 484 on Columbia agar plus 5% sheep blood (hemolytic activity experiment). After the incubation, 300 μL of 3% hydrogen peroxide solution was added to colonies and bubble formation was observed. The control was a catalase‐positive *S. aureus* strain (PCM 2054). The second method involved adding 1 mL of 3% hydrogen peroxide to a 2 mL overnight culture in LB broth in a glass tube and mixing thoroughly, followed by incubation at room temperature for 15 min. Oxygen bubbles and a foam column indicated positivity. No reagents were used to stop reactions. Bubbles in cultures also indicated catalytic activity. After reactions were completed, foam height, persisting for 20 min, was measured as previously described (Iwase et al. 2013).

### 2.7 Lactic Acid Production

Lactic acid production was determined using a l‐Lactic Acid Assay Kit (Magazyme). Assays were initiated by growing strain 484 and a probiotic reference strain *L. rhamnosus* GG (LGG, ATCC 53103). Liquid cultures were set up in 15 mL test tubes in 5 mL of medium and incubated for 24 h at 37°C (5% CO_2_). Next, tubes were centrifuged (4500 rpm for 8 min), with filtrates collected for assay in a 96‐well plate. A calibration curve was prepared using l‐Lactic Acid Standard reagent (5 mL, 0.15 mg/mL in 0.02% sodium azide). Test samples were diluted to 1:100.

### 2.8 Growth Independent Antagonistic Properties

Strain 484 antimicrobial properties were assayed against seven pathogens: *Staphylococcus epidermidis* NCIMB 8853, *Streptococcus mutans* ATCC 25157, *Streptococcus pyogenes* ATCC 49399, *E. coli* ATCC 25922, *E. coli* UPEC PCM 176, *Enterococcus faecalis* PCM 2784, and *Streptococcus agalactiae* PCM 2683. Strain 484 was inoculated into MRS broth and cultured for 24 h. Next, the culture was centrifuged, a pellet generated, and resuspended and diluted in sterile 0.85% NaCl to 0.5 McFarland (McF) units. Assays were conducted using a modified procedure (Choi et al. 2018). Pathogenic strains were inoculated into broth medium (Table 1) and similarly handled to strain 484. All strains were brought to 0.5 McF using a densitometer (DEN‐1B densitometer, Biosan) in sterile 0.85% NaCl. Strain 484 was mixed in a 1:1 ratio with each pathogen and co‐cultures incubated at 37°C for 1 h. Then, a series of dilutions was generated for each co‐culture. Simultaneously, single strain cultures were performed as controls on media appropriately selected for the pathogen. The serial dilution method was used to assess the CFU (colony forming units) count in each experiment. The reduction was calculated by comparing the pathogen CFU counts in co‐culture to those in single‐strain cultures (set as 100%).

**TABLE 1:** List of pathogens and growth medium and differentiation medium used to test antagonistic properties.

Pathogenic strain	ID	Source	Type	Patterning medium	Medium for antimicrobial properties assay
*Enterococcus faecalis*	2784	PCM Polish Collection of Microorganisms	Clinical strain	TSB (Biocorp)	Enterococosel Agar (Graso Biotech)
*Streptococcus mutans*	25,157	ATCC American Type Culture Collection	Reference	BHI (Biocorp)	Chromogenic medium for Strepto Base A (CHROMagar)
*Streptococcus pyogenes*	49,399	ATCC American Type Culture Collection	Reference	BHI (Biocorp)	Chromogenic medium for Strepto Base A (CHROMagar)
*Streptococcus agalactiae*	2683	PCM Polish Collection of Microorganisms	Clinical strain	BHI (Biocorp)	Chromogenic medium for Strepto Base A (CHROMagar)
*Escherichia coli* UPEC	176	PCM Polish Collection of Microorganisms	Clinical strain	LB (Biocorp)	UTI Agar (Himedia)
*E. coli*	25,922	ATCC American Type Culture Collection	Reference	LB (Biocorp)	Endo LAB Agar (Biocorp)
*Staphylococcus epidermidis*	8853	NCIMB National Collection of Industrial, Food and Marine Bacteria	Clinical strain	TSA (Biocorp)	Chromogenic medium for Staphylococcus (CHROMagar)

### 2.9 Antibiotic Sensitivity

To evaluate safety, antibiotic susceptibility was performed using the disc diffusion method on MRS agar (Biomaxima) as described (Chen et al. 2020). Strain 484 cultures were spread onto MRS agar plates and dried before disc placement. Antibiotic discs were: Amoxicillin 20 + Clavulanic acid 10 (Mast Diagnostics), Clindamycin 2 (Biomaxima), Piperacillin 30 (Biomaxima), Piperacillin 30 + Tazobactam 6 (Mast Diagnostics), Ticarcillin 75 (Mast Diagnostics), Ticarcillin 75 + Clavulanic acid 10 (Mast Diagnostics), and Tigecycline 15 (Mast Diagnostics). Discs were evenly placed on agar surfaces and plates incubated at 37°C for 24 h under aerobic conditions. Transparent zone (mm) diameters around discs were then used to classify strains as resistant, intermediate, or susceptible according to EUCAST regulations.

### 2.10 Cell Surface Hydrophobicity (CSH)

Cell surface hydrophobicity (CSH) was determined by inoculating liquid MRS broth (Biomaxima) with the strain 484 and incubating for 24 h at 37°C with constant shaking (180 rpm). A control *L. rhamnosus* GG (LGG, ATCC 53103) culture was also established. After 24 h, cultures were washed three times with distilled water to remove residual medium, and cells were resuspended in distilled water to achieve approximately 4° on the McFarland scale. Suspensions were then applied to microscope slides, which were incubated at 37°C until the water had completely evaporated and an even bacterial layer remained. Contact angles of distilled water (2 μL droplets) were measured using an OCA 15EC contact angle goniometer (DataPhysics Instruments) at room temperature. Assays were performed in triplicate, with at least three serial measurements per replicate, resulting in a total of *n* = 10 measurements. One‐way ANOVA was used to assess the statistical significance of the results.

### 2.11 Biofilm Staining

To assess whether strain 484 adhered to surfaces and formed biofilms, and to visualize cell morphology, live/dead staining was performed using the FilmTracer kit (Invitrogen). Microscopy cover slides were placed into 50 mL tubes filled with 15 mL of MRS broth and inoculated with an overnight strain culture at a 1:100 ratio. Tubes were incubated at 37°C for 7 days, after which slides were washed in sterile PBS and stained with a mixture of SYTO9 and propidium iodide (FilmTracer, Invitrogen) in the dark. Cover slides were then mounted onto microscope slides, representative images captured using an Axio Scope.A1 epifluorescence microscope (Carl Zeiss), and finally superimposed using ImageJ software (https://imagej.net/ij/index.html) and the Z‐stack tool (Max‐intensity algorithm).

### 2.12 Biomass Production

To evaluate biomass production by strain 484, five commercially available microbiological media recipes were prepared and tested. Media composition is listed (Table 2). Each medium was prepared in 100 mL in round‐bottom flasks and sterilized at 121°C for 12 min. Then, a 1 mL inoculum containing 1.3 × 10^8^ colony forming units (CFU)/mL was added to each flask. Cultures were incubated for 24 h at 37°C with continuous shaking. After this, viable cell numbers were determined using pour plate and serial dilution methods. Potential biomass production was then assessed by scale‐up in a Sartorius BiostatC bioreactor (20 L working volume) using a Rushton turbine (150 rpm at 37°C). A constant airflow was maintained at 5 L/min (at 4 bar) for 24 h and the inoculation volume was 200 mL.

**TABLE 2:** List of components used as ingredients in media tested for production scale of *Lacticaseibacillus hamnosus* 484 biomass production.

Component	Medium 1	Medium 2	Medium 3	Medium 4	Medium 5
Food‐grade whey powder	5%	5%	5%	5%	2.5%
Phosphoric acid (pH 3)	Yes	Yes	—	—	Yes
Pepsin	0.02% (with 37°C/30 min incubation)	0.02% (with 37°C/30 min incubation)	—	—	0.01% (with 37°C/30 min incubation)
Ammonia solution (25%) (pH 7)	Yes	Yes	—	—	Yes
Yeast extract	0.5%	—	—	—	—
Dried yeast	—	2.0%	2.0%	—	2.0%
Maltodextrin	—	—	—	0.5%	—
Inulin	—	—	—	0.5%	—

### 2.13 Whole Genome Sequencing and De Novo Assembly

DNA was isolated using a Genomic Mini A Bacteria+ kit (A&A Biotechnology) and resuspended in TE buffer. DNA concentrations were measured using a Quant‐iT PicoGreen dsDNA Assay Kit (Thermo Scientific) and assessed by electrophoretic separation on a 1% agarose gel plus a DNA Lambda/HindIII marker. Next, library preparation and sequencing were initiated by fragmenting genomic DNA via sonication (Covaris E210, Covaris) according to parameters recommended for preparing libraries for sequencing using Illumina technology. Libraries were prepared using the NEBNext Ultra II DNA Library Prep Kit for Illumina (New England Biolabs, E7645L). Libraries for the MinION device were prepared using the Rapid Barcoding Kit (SQK‐RBK004). Libraries were sequenced on a MinION instrument with a Flongle R10.4.1 Standard flow plate. Assembly analysis was performed using the Galaxy program (Galaxy Version 0.5.0 + galaxy1) with the Unicycler tool (Wick et al. 2017) and the SPAdes algorithm (default settings). Genome annotation was performed using the Bakta Web program (Software: 1.9.1 | DB: 5.0.0) (Schwengers et al. 2021) and the Prokaryotic Genome Annotation Pipeline (online version) (Tatusova et al. 2016).

### 2.14 Screening for Mobile Genetic Elements (MGEs), Antimicrobial Resistance Genes (ARGs), and Secondary Metabolites

Plasmids in assembled sequences were determined using PlasmidFinder 2.1 (Software version: 2.0.1: 2020‐07‐01, database version: 2023‐01‐18, minimum 95% identity and minimum 60% coverage) (Carattoli et al. 2014) and MGEs using MobileElementFinder (Software version: v1.0.3 [2020‐10‐09], Database version: v1.0.2 [2020‐06‐09]) (Johansson et al. 2021). MGE sequences from MobileElementFinder were analyzed using the Comprehensive Antibiotic Resistance Database (CARD 3.2.9) through the Resistance Gene Identifier (RGI 6.0.3) tool (Alcock et al. 2020) to identify antimicrobial resistance genes (ARGs). The following parameters were used: *Data Type*: DNA sequence; *Criteria*: Perfect and Strict hits only, with nudge ≥ 95% identity for Loose hits to Strict; *Sequence Quality*: High quality/coverage. Additionally, sequences were analyzed using ResFinder (software version: 2024‐03‐22; database version: 2024‐03‐22) (Florensa et al. 2022). The PHAge Search Tool Enhanced Release (PHASTEST) (Wishart et al. 2023) as well as VirSorter (Proksee) (Grant et al. 2023) were used to identify prophages in the genome. The assembly was also examined for Clustered Regularly Interspaced Short Palindromic Repeats (CRISPR) arrays using CRISPRCasFinder (online version; default settings) (Couvin et al. 2018). Secondary metabolites were assessed using the antiSMASH on‐line tool (version 8.0.1) using strict settings (Blin et al. 2025).

### 2.15 Phylogenetic Analysis

Orthologous fragments from the strain 484 were compared to the *L. rhamnosus* 1.0320 reference strain using the EZBioCloud platform (EZBioCloud Database, Update 2023.08.23) and the OrthoANI algorithm, which uses USEARCH (Yoon et al. 2017). A phylogenetic tree was constructed by analyzing single nucleotide polymorphisms (SNPs) in the CSI Phylogeny tool (Kaas et al. 2014). The “newick file” was uploaded to the iTOL service to construct a phylogenetic tree (Letunic and Bork 2024). Phylogenetic analysis was performed using 48 random *L. rhamnosus* genomes from the National Center for Biotechnology Information (NCBI) database, along with the reference genome (strain 1.0320) and 484 strain genome.

### 2.16 Genome Accession Number

The whole‐genome shotgun project was submitted to the NCBI GenBank database (accession number = CP174089).

## 3 Results

### 3.1 Auto‐ and Co‐Aggregation

**FIGURE 1:**
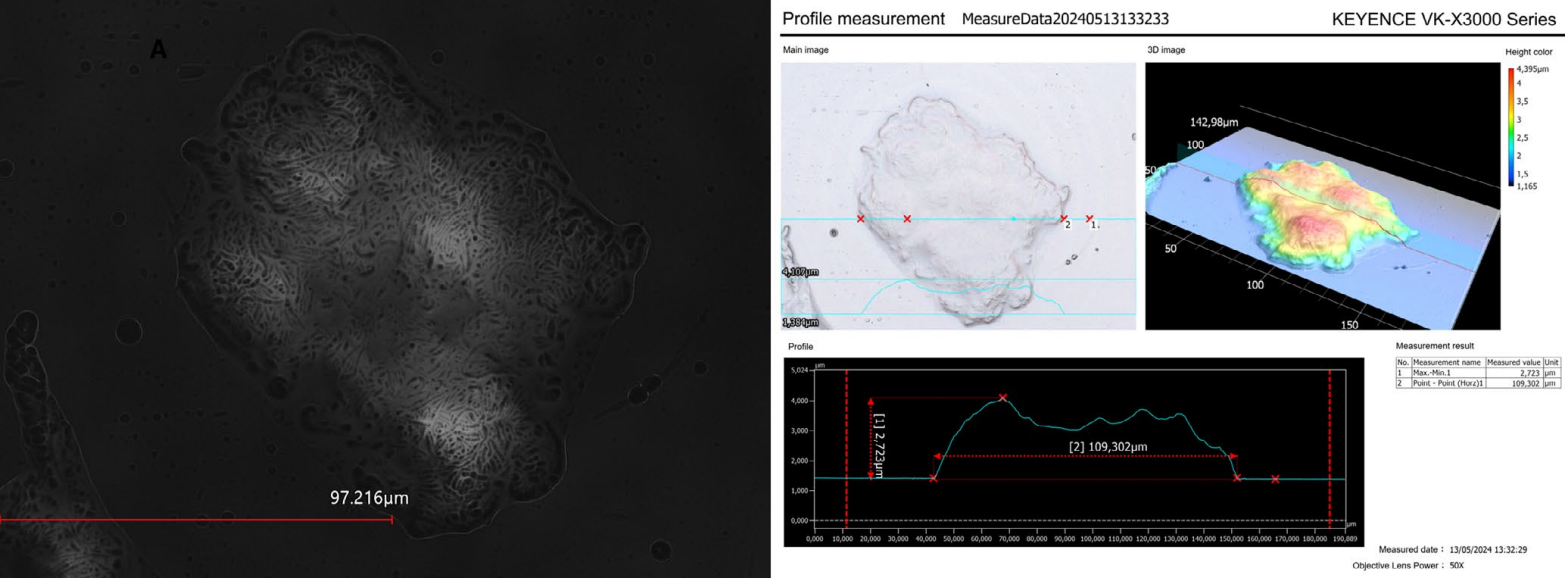
Microphotograph of auto‐aggregates produced by *Lacticaseibacillus rhamnosus* strain 484 during storage in the form of a prototype preparation for use as a dermatological product was visualized using VK‐X3000 confocal microscopy. The scale bar represents the diameter of the aggregate.

Self‐aggregation is a survival strategy that has significant physiological implications for host health and microecological balance. This phenomenon, a LAB characteristic, has crucial roles inhibiting environmental pathogens. To evaluate aggregation in strain 484, both auto‐ and co‐aggregation assays were performed, with aggregates (clumps) analyzed by confocal microscopy. Auto‐aggregation results indicated that strain 484 had an auto‐aggregation ability of 15.38% ± 0.014%. Additionally, a co‐aggregation ability of 57.65% ± 0.003% was determined for *E. coli* PCM176 (pathogenic strain). Aggregates were irregular and clumped, forming three‐dimensional structures. The clusters were non‐uniform, with an average size of approximately 100 μm (Figure 1).

### 3.2 Tolerance to Bile Salts, Resistance to Low pH, and Lactic Acid Production

To test strain 484 tolerance to bile salts, cells were cultivated at 37°C for 24 h in MRS medium plus different bile salt concentrations: 0.1%, 0.3%, 0.5%, and 1.0%. Cells were viable on medium containing bile salts in the 0.1% to 1.0% concentration range. Additionally, to assess acid tolerance, cells were incubated at 37°C for 24 h in MRS medium adjusted to varying pH's (2.0, 3.0, and 4.0). Cells grew at pH 3.0 and above; however, no growth was observed at pH 2.0. Moreover, strain 484 showed significantly higher lactic acid production (14.11 mg/mL) levels when compared to the LGG strain (12.89 mg/mL).

### 3.3 Hemolytic Activity and Catalase Tests

Strain 484 was tested for hemolytic activity. Typically, hemolytic *α* and *β* activity disqualifies bacterial strains from being considered safe and excludes their probiotic potential. *S. aureus* , which exhibits β‐hemolysis, was used as a positive control. Strain 484 and *S. aureus* were cultured for 48 h on solid medium containing sheep blood, after which cultures were examined. No *α* or *β* hemolytic activity was observed for strain 484. The strain was also tested for catalytic activity, a property that disqualifies bacterial strains in terms of safety and excludes their probiotic potential. *S. aureus* was used as a positive control. Both strain 484 and *S. aureus* were cultured for 48 h on solid medium containing sheep blood, after which colonies were subjected to a 3% hydrogen peroxide solution. The appearance of air bubbles indicated catalase activity. Strain 484 did not produce air bubbles, confirming it as catalase‐negative. In the second method, 1 mL of 3% hydrogen peroxide was added to 2 mL of bacterial culture in a tube. A vigorous reaction was observed for the *S. aureus* strain—oxygen bubbles were generated and a foam column measured 120 mm. In contrast, no catalase activity was observed for strain 484.

### 3.4 Growth Independent Antagonistic Properties

The antibacterial properties of strain 484 were evaluated against seven pathogens: *S. epidermidis* NCIMB8853, *S. mutans* ATCC25157, *S. pyogenes* ATCC49399, *E. coli* ATCC25922, *E. coli* UPEC PCM176, *E. faecalis* PCM2784, and *S. agalactiae* PCM2683. Bacterial cultures were grown on solid media. The reduction in pathogenic strains varied, but in all cases, it exceeded 50%, except *S. epidermidis* NCIMB8853, which showed a reduction of 40.65% ± 10.39%. The highest inhibition was observed for *E. coli* ATCC25922, which showed 84.30% ± 3.536%. Pathogen inhibition results are shown (Table 3).

**TABLE 3:** The percentage reduction of each pathogenic strain by the *Lacticaseibacillus rhamnosus* 484 strain, along with the standard deviation of each result.

Pathogen	Strain ID	Reduction (%)	Standard deviation (±)
*Staphylococcus epidermidis*	NCIMB8853	40.65	10.394
*Streptococcus mutans*	ATCC25157	66.60	1.131
*Streptococcus pyogenes*	ATCC49399	81.60	2.899
*Escherichia coli*	ATCC25922	84.30	3.536
*E. coli* UPEC	PCM176	54.70	1.485
*Enterococcus faecalis*	PCM2784	73.60	15.486
*Streptococcus agalactiae*	PCM2683	51.40	9.192

### 3.5 Cell Surface Hydrophobicity and Biofilm Formation

**FIGURE 2:**
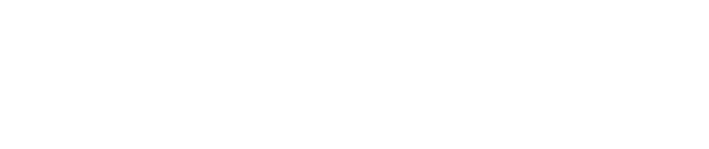
Representative images illustrating water droplet shape on bacterial layer. Cell surface hydrophobicity is expressed as the contact angle of a water droplet resting on layers composed of (A) *Lacticaseibacillus rhamnosus* 484, (B) *L. rhamnosus* GG, and (C) control—microscope glass slide. Images were acquired using the DataPhysics OCA 15 (OCA 15 EC) contact angle goniometer equipped with a 6.5× zoom lens (providing up to 6.5‐fold magnification of the droplet image).

**FIGURE 3:**
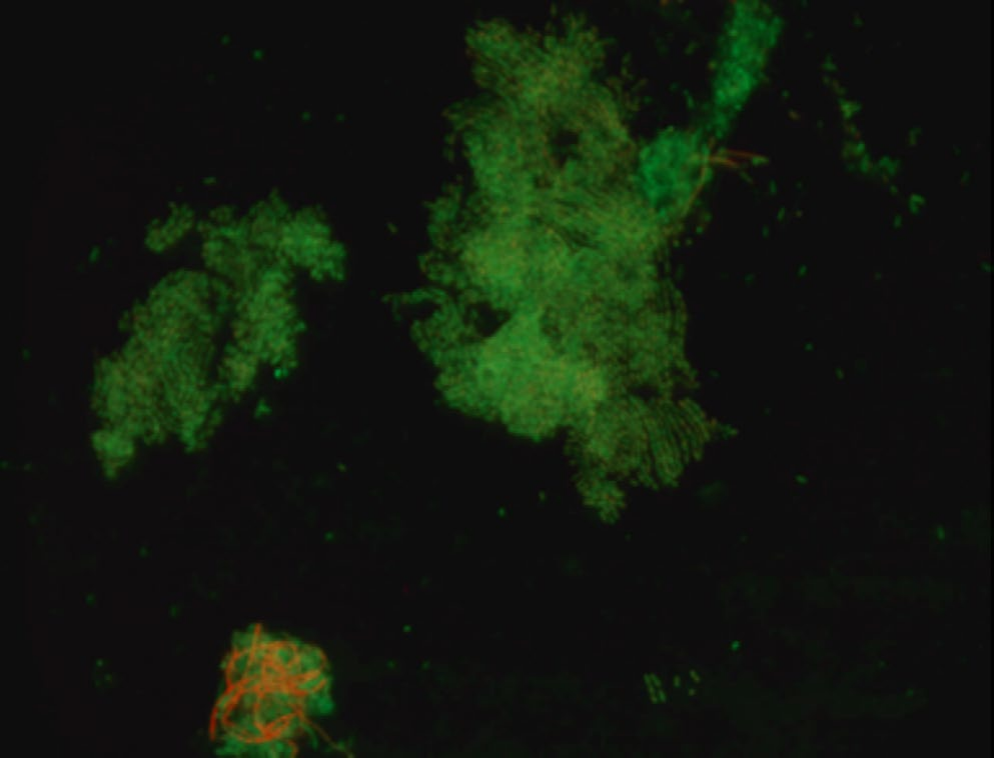
Image of 7‐day old biofilm of *Lacticaseibacillus rhamnosus* strain 484 stained with the Live/Dead viability assay (FilmTracer) where dead are represented in red due to membrane damage and penetration by the propidium iodide dye. Live cells are marked by green Syto9 stain. The image was generated with an Axioscope.A1 microscope (Zeiss) with total magnification of ×1000.

Contact angle measurements were performed to assess CSH for strain 484, the LGG strain, and a glass control. Measurements were taken at both left and right angles for each sample. Strain 484 had an average contact angle of approximately 90.07° (left) and 90.03° (right), with standard deviations of 8.36 and 8.21, respectively (indicating low wettability). For the test LGG strain, a reduced hydrophobicity was observed when compared to strain 484, resulting in average contact angles of 74.12° (left) and 74.14° (right), and standard deviations of 8.21 and 8.00, respectively. Soda‐lime glass (microscopic slides) was used as a negative control, which showed the lowest contact angles: 39.04° (left) and 39.02° (right), with standard deviations of 9.54 and 8.81, respectively, indicating high wettability (Table 4, Figure 2). These results suggested that strain 484 had the highest CSH, followed by LGG, and the glass control having the most hydrophilic properties. Variable standard deviation values across samples potentially reflected differences in surface interactions. Statistical analyses showed that strain type had a highly significant impact on hydrophobicity (*p* < 0.001), while left versus right contact angles did not significantly differ (*p* > 0.05). Surface adhesion and biofilm formation evaluations were performed using live/dead staining on 7‐day‐old strain 484 biofilms on soda‐lime glass by microscopy. Image analysis revealed dead cells (stained red), although live cells (stained green) predominated. Cell morphology was characteristic of bacterial biofilm microcolonies. Representative images are shown (Figure 3).

**TABLE 4:** Cell surface hydrophobicity results expressed as the contact angle of the tangent to water droplet settled on the surface of the bacterial layer.

Strain	484		LGG		Control (glass)	
	Left angle	Right angle	Left angle	Right angle	Left angle	Right angle
Average (°)	90.07	90.03	74.12	74.14	39.04	39.02
Standard deviation	8.36	8.21	8.21	8.00	9.54	8.81

*Note:* To assess the statistical significance of differences in cell surface hydrophobicity (measured via contact angle) across strains and angle sides, a two‐way ANOVA was performed using *n* = 10.

### 3.6 Genome Overview

To gain insights into the genetic makeup of strain 484, we performed a comprehensive genome annotation. The analyses provided a detailed overview of functional and regulatory elements present in the genome. Annotation analysis using the Bakta Web platform provided an overview of the various genetic elements in strain 484's genome. The presence of 59 transfer RNA genes suggested molecules that recognized different codons, which are required for efficient and accurate protein synthesis. A single transfer‐messenger RNA gene had a dual function: rescuing stalled ribosomes on damaged microRNA and tagging incomplete polypeptides for degradation. The identification of 15 ribosomal RNA (rRNA) genes typically reflects multiple rRNA operon copies, thereby ensuring adequate ribosome production to meet cellular demands. Non‐coding RNAs, which are implicated in various regulatory functions, such as gene expression control and RNA processing, were represented by four genes. This indicated regulatory complexity within strain 484's genome; however, further functional analyses are required to determine specific roles. Additionally, 23 non‐coding RNA regions suggested substantial non‐coding functionality, potentially involved in post‐transcriptional regulation. Annotation analyses also revealed a CRISPR region, which potentially provided adaptive immunity against foreign genetic elements, such as bacteriophages, and allowed the host to recognize and degrade DNA from invaders. The presence of 2811 coding DNA sequences indicated the protein‐coding potential of the genome and represented genes that encoded various enzymes, structural proteins, and other functional molecules required for cellular processes. Additionally, five small open reading frames (sORFs) were identified. These shorter coding sequences potentially encoded small proteins or peptides, with specialized or regulatory roles in the cell, although they were less characterized than standard coding sequences. A single origin of chromosomal replication was also identified, while no plasmid origin of replication (*oriV*) was found, suggesting that the genome was plasmid‐free or lacked endogenous plasmid replication elements. Furthermore, the absence of an origin of transfer indicated that the genome may have lacked conjugative elements for horizontal gene transfer, such as conjugative plasmids or transposons. Finally, no gaps were identified in the assembly, indicating a complete and continuous genome sequence with no missing regions.

### 3.7 Antimicrobial Resistance

Strain 484's genome was analyzed using two databases (CARD/RGI and ResFinder) and focused on antimicrobial resistance genes (ARGs) detection, but no known ARGs were identified. To verify this, classic antibiograms were generated using selected antibiotics, with antibiotics representing cell wall synthesis and protein synthesis inhibitors (Table 5). Strain 484 showed sensitivity to four antibiotics belonging to the cell wall synthesis inhibitor group. The largest growth inhibition zone, as much as 35 mm, was visible for the ticarcillin and clavulanic acid (TIM85C) combination. Equally, large inhibition zones were identified for other antibiotics in this group and piperacillin belonging to β‐lactams. Strain 484 has been classified as susceptible to these antibiotics, indicating efficacious β‐lactamase inhibition in overcoming bacterial resistance mechanisms. A similar observation was recorded for two antibiotics belonging to the protein synthesis inhibitor group. The first was observed in the glycylcycline subgroup—tigecycline, and the other clindamycin is lincosamide. Here, growth inhibition zones were 27 and 13 mm, respectively, which, according to *Enterobacterales* and *Bacillus* species standard guidelines, classified strain 484 as susceptible to both antibiotics.

**TABLE 5:** The study investigated the efficacy of various antibiotics with different modes of action against *Lacticaseibacillus rhamnosus* 484.

Group	Antibiotic (dose μg/mL)	Acronym	Strain 484—zone [mm]	Sensitivity by standards for *Enterobacterales* and *Bacillus*	EUCAST *(according to Enterobacterales/Bacillus)*
Inhibitors of cell wall synthesis					
Penicillins	Piperacillin 30	*PRL30*	26	Sensitive	*Enterobacterales* (S >/= 20; *R* < 20)^a^
Penicillins with β‐lactamase inhibitor	Amoxicillin 20 + Clavulanic acid 10	*AUG30C*	24	Sensitive	*Enterobacterales* (S >/= 19^; *R* < 19^; ATU 19–20)^a^
	Piperacillin 30 + Tazobactam 6	*PTZ36C*	29	Sensitive	*Enterobacterales* (S >/= 20; *R* < 20; ATU 19)^a^
	Ticarcillin 75 + Clavulanic acid 10	*TIM85C*	35	Sensitive	*Enterobacterales* (S >/= 23; *R* < 20)^a^
Inhibitors of protein synthesis					
Glycylcyclines	Tigecycline 15	*TGC15C*	27	Sensitive	*Enterobacterales* (S >/= 18^A,B; *R* < 18^A,B) ( *E. coli* and *C. koseri* )^b^
Lincosamides	Clindamycin 2	*DA2*	13	Sensitive	*Bacillus* spp. (S >/= 17; *R* < 17)^c^

*Note:* Each antibiotic was tested at a specific concentration (expressed in μg/mL), and its effectiveness was determined by measuring the diameter of the growth inhibition zone (in millimeters). Based on standard guidelines for *Enterobacterales* and *Bacillus* species, the susceptibility of *L. rhamnosus* 484 to the tested antibiotics was evaluated, allowing the strain to be classified as susceptible.

^a^ Coppola et al. (2005).

^b^ Bartalesi et al. (2012).

^c^ Charteris et al. (1998) and Coppola et al. (2005).

### 3.8 Plasmids, MGEs, and CRISPR Sequences

**FIGURE 4:**
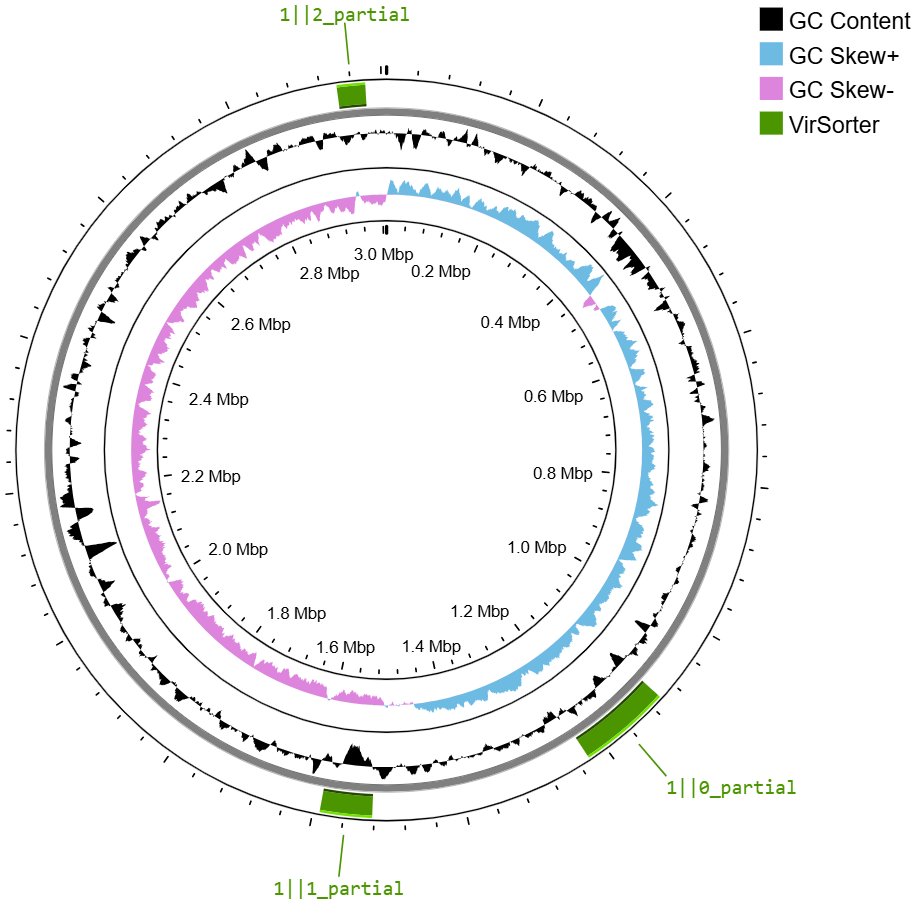
Circular map representing prophage regions in *Lacticaseibacillus rhamnosus* 484 genome. Prophages were recognized as sipho‐type viruses, detected both by VirSroter (Proksee) and confirmed by the PHASTEST tool.

Mobile genetic elements (MGEs), plasmids, and CRISPR‐Cas systems are key determinants of genome plasticity, stability, and defense against foreign DNA. Therefore, their presence and organization in strain 484 were investigated in detail. According to the Bakta Web annotation platform, no *oriV* sequence was identified, and no plasmids were detected (PlasmidFinder2 tool). However, 40 MGEs were identified. In this dataset, MGEs were analyzed by aligning to reference sequences, focusing specifically on insertion sequences (ISs) and composite transposons. MGE analyses identified ISLrh4, ISLrh2, and ISLrh3 ISs, which are transposable elements containing a transposase gene flanked by short terminal inverted repeats. ISLrh4 was found at five locations, with copies showing 100% sequence identity and coverage, indicating perfect matches to the reference sequence. ISLrh2, detected at 22 locations, showed a high sequence identity of approximately 99%. ISLrh3 was identified at two locations. Additionally, two composite transposon types, which consisted of two ISs flanking one or more functional genes, were detected. A composite transposon, cn_16187_ISLrh4, was identified, as well as a large composite transposon, cn_36734_ISLrh2, inferred from the ISLrh2 element. Furthermore, several other composite transposons, ranging in size from 5326 to 49,798 bp, were identified based on ISLrh2, suggesting the widespread distribution and potential mobilization of these elements. All MGEs are listed (Table S1). Additionally, CRISPR sequences were found in strain 484's genome. The Cas cluster Type IIA was found with four spacer elements, and also a CRISPR sequence 5′‐GCTCTTGAACTGATTGATCTGACATCTACCTGAGAC‐3′ separated by 23 spacer elements. A prophage search (PHASTEST and VirSorter tools) also revealed three prophage regions, with prophages recognized as sipho‐type virus (former *Siphoviridae* family) (Figure 4).

### 3.9 Phylogenetic Analysis

**FIGURE 5:**
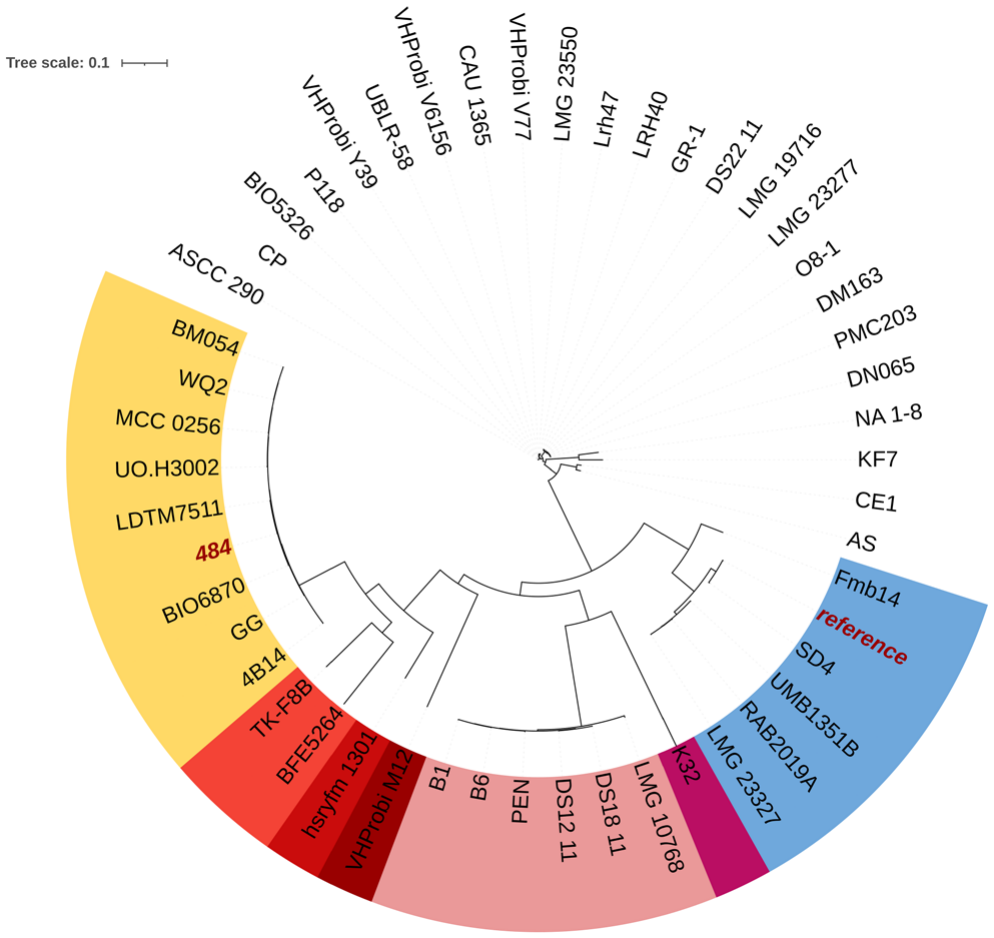
Phylogenetic tree of *Lacticaseibacillus rhamnosus* genomes. The tree was constructed based on SNP analysis of 50 *L. rhamnosus* genomes. Labels are color‐coded according to clades, representing closely related isolates. Reference strain: *L. rhamnosus* 1.0320. Both *L. rhamnosus* 484 and reference strains were highlighted in red.

A phylogenetic analysis was performed to compare strain 484's genome with a reference genome ( *L. rhamnosus* 1.0320 strain). The OrthoANI value showed 97.30% identity between strains (Table 6). To evaluate other closely related strains, SNP analysis was performed, and a phylogenetic tree was created (Figure 5). Strain 484 was closely related to the LGG strain, a widely used probiotic. From SNP analyses, other closely related strains included 4B14, BIO6870, LTDM7511, UO.H3002, MCC 0256, WQ2, and BM054. These strains, along with strain 484, formed a distinct evolutionary lineage.

**TABLE 6:** OrthoANI calculator results of comparison of *Lacticaseibacillus rhamnosus* 1.0320 and *L. rhamnosus* 484 strain.

Metric	Value
OrthoANIu value (%)	97.30
Reference genome length (bp)	2,940,660
Genome 484 length (bp)	3,006,960
Average aligned length (bp)	1,936,074
Genome 1.0320 coverage (%)	65.84
Genome 484 coverage (%)	64.39

### 3.10 Secondary Metabolite Analyses

To explore the secondary metabolite biosynthetic potential of strain 484, its genome was analyzed (antiSMASH tool). Although not a typical prolific producer of secondary metabolites like *Streptomyces* or *Burkholderia*, several biosynthetic gene clusters were identified in strain 484 and showed low to moderate similarity to known biosynthetic clusters, suggesting a latent capacity for specialized metabolite production. Recognized clusters are listed in Table 7.

**TABLE 7:** Summary of biosynthetic gene clusters (BGCs) identified in *Lacticaseibacillus rhamnosus* 484 using antiSMASH tool.

BGC ID	Cluster type	Predicted product (s)	Similarity score	Reference organism	Predicted function
BGC0000866.5	Other (PHA)	Polyhydroxyalkanoate	0.47	Burkholderia sp. DSM 9242	Biopolymer biosynthesis for energy storage and stress resistance
BGC0000867.3	Other (PHA)	Polyhydroxyalkanoic acids	0.46	*Ectothiorhodospira shaposhnikovii*	Redox balance and carbon storage
BGC0002362.3	PKS (Type I)	Loseolamycin A1, A2	0.42	*Micromonospora endolithica*	Polyketide antibiotics with potential antimicrobial activity
BGC0000554.4	Ribosomal (RiPP)	SRO15‐3108	0.40	*Streptomyces filamentosus* NRRL 15998	Antimicrobial peptide (possibly bacteriocin‐like)
BGC0002561.3	Other (Phenazine)	Phenazine SA, SB, SC	0.40	Streptomyces sp.	Redox‐active compounds with antimicrobial potential
BGC0001521.3	NRPS	Auriculamide	0.39	*Herpetosiphon aurantiacus* DSM 785	Cyclic peptide with potential cytotoxic or signaling activity
BGC0001591.4	Other	Fatty acid enol ester, N‐tetradecanoyl tyrosine	0.38	Uncultured bacterium CSLC2	Membrane‐active or quorum‐sensing related metabolites
BGC0001151.5	Other (non‐NRP β‐lactam)	Valclavam, (‐)‐2‐(2‐hydroxyethyl)clavam	0.38	*Streptomyces antibioticus*	β‐lactamase inhibitors with possible synergistic antibiotic potential
BGC0001080.5	Other (Phenazine)	Endophenazine A, B	0.37	*Streptomyces anulatus*	Phenazine derivatives with antimicrobial activity
BGC0000286.3	PKS	Viguiepinol	0.36	Streptomyces sp. KO‐3988	Aromatic polyketide with potential antifungal activity

### 3.11 Biomass Production

To evaluate strain 484 potential as a probiotic, five commercially available culture medium compositions were prepared and biomass assays conducted. For each medium, the same initial inoculum (1.3 × 10^8^ CFU, ratio 1:100) was used. After 24 h, final bacterial concentrations varied significantly between media, indicating differences in bacterial growth. Medium 1 showed the highest growth, reaching 1.0 × 10^10^ CFU/mL after 24 h incubation, while Medium 4 showed the lowest at 1.5 × 10^9^ CFU/mL. Medium 2 and 5 demonstrated identical growth performances, and Medium 3 exhibited moderate growth. This final media was the simplest and most cost‐effective to prepare. For this reason, scale‐up was performed using Medium No. 3 in a bioreactor. Consequently, by maintaining aeration (5 L/min at 4 bar) and agitation (150 rpm), a density of 3.0 × 10^9^ CFU/mL was generated after 24 h.

## 4 Discussion

HBM fulfills the nutritional requirements of newborns and infants while playing a critical role in shaping the immune system, thereby providing protection against pathogens. It is rich in a diverse array of bioactive molecules, including immunocompetent cells, immunoglobulins, fatty acids, polyamines, oligosaccharides, antimicrobial peptides, and probiotics (Fernández et al. 2013). Recent studies suggest that lactobacilli isolated from HBM represent an effective alternative to antibiotics (Obisesan et al. 2024). Furthermore, *Lactobacillus* strains derived from breast milk have been shown to enhance both the innate and adaptive immune responses by activating NK cells, stimulating T cells, and promoting the expansion of regulatory T cells (Fontana et al. 2013). *L. rhamnosus* strains show strong potential in controlling pathogenic bacteria across clinical, industrial, and environmental settings. Their antimicrobial efficacy is attributed to the production of organic acids, hydrogen peroxide, and bacteriocins, including bacteriocin‐like inhibitory substances (BLIS), which inhibit a wide range of pathogens (Martinez et al. 2015; Mousavi Khaneghah et al. 2020). Additionally, *L. rhamnosus* competes with pathogens for adhesion sites on epithelial surfaces, reducing infection risk (Alonso‐Roman et al. 2022), and demonstrates immunomodulatory effects that enhance host defense (De Keersmaecker et al. 2006; McFarland et al. 2018).

The aim of this study was to evaluate the probiotic characteristics of a newly isolated *L. rhamnosus* strain 484, derived from HBM, and assess its potential as a novel probiotic agent. *L. rhamnosus* 484 exhibited strong antagonistic activity, particularly against *E. coli* ATCC 25922 (84.3% inhibition), *S. pyogenes* (81.6%), *S. mutans* (66.6%), and *E. faecalis* (73.6%), suggesting broad‐spectrum antimicrobial potential. These effects were likely mediated by antimicrobial metabolite production. Another key probiotic trait of strain 484 was its high CSH, which supported adhesion, colonization, and biofilm formation, critical factors for persistent probiotic activity. A contact angle slightly above 90° confirmed its non‐wettable surface, which has been linked to enhanced aggregation, co‐aggregation, and resistance to displacement in the gut (Durán and Laroche 2019; Polak‐Berecka et al. 2014). The strain 484 displayed a significantly higher CSH when compared to the widely used *L. rhamnosus* GG (LGG) strain, suggesting its high potential to colonize intestinal epithelium. Moreover, surface properties are essential for interactions with host tissues and the competitive exclusion of pathogens. Comparative studies have also confirmed that strains like *L. rhamnosus* CWKu‐12, *Lactococcus lactis* NJ414, and *Levilactobacillus brevis* Lb13H shared similar probiotic traits, including antimicrobial activity, high aggregation, and biofilm inhibition properties (Alizadeh Behbahani et al. 2024; Echresh et al. 2024; Rahmati‐Joneidabad et al. 2024). These findings support the evaluation of newly isolated strains in terms of safety and functionality profiles. Other established probiotic strains, such as *Lactobacillus acidophilus* NCFM, *L. reuteri* DSM 17938, *L. johnsonii* La1, *L. plantarum* 299v, and *L. casei* Shirota, have provided context for examining the probiotic potential of strain 484, which demonstrated comparable properties, including bile salt resistance, antimicrobial activity, and host interaction capabilities.

Whole‐genome sequencing confirmed no ARGs and virulence factors in strain 484, while no plasmids were detected. MGEs typical of *L. rhamnosus* were present, but no concerning features were identified (Moradi et al. 2022). SNP analyses revealed high genetic similarity to well‐characterized probiotic strains, such as LGG, BIO6870, and LDTM 7511, further supporting the safety and potential of strain 484 (Petraro et al. 2024; Yeo et al. 2022). To estimate the potential of secondary metabolite synthesis, the antiSMASH analysis of *L. rhamnosus* 484 was performed and showed the presence of biosynthetic gene clusters related to the production of nonribosomally synthesized antimicrobial metabolites. These metabolites are typically assembled by large enzyme complexes such as nonribosomal peptide synthetases (NRPSs) or polyketide synthases (PKSs). Unlike ribosomally derived bacteriocins, these compounds often exhibit structural diversity and broader bioactivity spectra, enabling them to act against both Gram‐positive and Gram‐negative bacteria (Blin et al. 2025; Medema and Fischbach 2015). The mechanisms of inhibition are varied but commonly include disruption of cell membrane integrity, interference with peptidoglycan biosynthesis, or inhibition of essential enzymatic processes (Cimermancic et al. 2014). Moreover, many NRPS/PKS‐derived metabolites act at very low concentrations and can bypass some resistance mechanisms that pathogens employ against conventional bacteriocins or antibiotics (Marahiel 2016). Other studies have reported that certain secondary metabolites produced by the gut bacteria inhibit *Clostridioides difficile* spore germination (Gurung et al. 2024) which may provide useful insights for strain 484 as a probiotic targeting *C. difficile* infection. Thus, the predicted biosynthetic capacity of strain 484 highlights that its antimicrobial action is unlikely to rely solely on organic acids or hydrogen peroxide. Instead, *L. rhamnosus* 484 may employ a multifaceted inhibition strategy, combining metabolic byproducts (lactic acid, acetic acid, H_2_O_2_) with structurally diverse secondary metabolites of NRPS/PKS origin, which would explain its pronounced inhibitory potential against diverse pathogens. Additionally, pilot‐scale cultivation studies further confirmed strain 484's industrial potential. Medium composition significantly affected biomass yields, with pepsin and yeast extract (Medium 3) producing the best results. These findings aligned with studies emphasizing tailored media in optimizing probiotic growth and metabolite production (Choi et al. 2021).

## 5 Conclusions

Our study highlighted the potential benefits of a probiotic strain of *L. rhamnosus* derived from HBM, indicating that HBM is a promising source of beneficial bacteria. Through comprehensive genomic and phenotypic analyses, we gained valuable insights into the genetic composition and the probiotic properties of strain 484. Specifically, the strain possessed promising characteristics, including the ability to inhibit pathogenic bacterial growth via carbon source‐independent mechanisms. An in silico safety assessment revealed that strain 484 lacked ARGs and virulence factors, confirming its safety for human use. Moreover, the strain exhibited strong probiotic potential, evidenced by its stress resistance, adhesion capacity, and the ability to form biofilms. Notably, strain 484 displayed a significantly higher CSH when compared to the widely used *L. rhamnosus* GG (LGG) strain. Future research must examine the mechanisms underlying this carbon source‐independent antibacterial activity and explore its role in preventing pathogenic bacterial spread. Further studies should also investigate the strain‐specific properties, mechanisms of action, and synergistic effects of *L. rhamnosus* strains in combination with other probiotics or therapeutic agents. These efforts may optimize their applications in targeted interventions, broaden their utility, and enhance their efficacy across diverse clinical and industrial settings.

## Author Contributions

**Natalia Czarnecka:** conceptualization (equal), funding acquisition (equal), investigation (equal), methodology (equal), project administration (equal), writing – original draft (equal), writing – review and editing (equal). **Magdalena Jankowska:** conceptualization (equal), data curation (equal), investigation (equal). **Sylwia Nawrot:** investigation (equal). **Katarzyna Nogal‐Nowak:** data curation (equal). **Sławomir Wąsik:** investigation (equal). **Grzegorz Czerwonka:** conceptualization (equal), formal analysis (equal), funding acquisition (equal), investigation (equal), methodology (equal), project administration (equal).

## Ethics Statement

All methods were conducted in full compliance with relevant guidelines and regulations, adhering to the confidentiality and ethical principles outlined in the Declaration of Helsinki.

## Consent

Bacterial samples were isolated from patients after obtaining informed consent from all participants, in accordance with the guidelines of the Bioethics Committee at the Świętokrzyska Medical Chamber, as specified in Resolution No. 21/2020 VII (Kielce, September 4, 2020).

## Conflicts of Interest

The authors declare no conflicts of interest.

## Supporting information


**Data S1:** fsn370980‐sup‐0001‐Supinfo.png.

**Data S2:** fsn370980‐sup‐0002‐TableS1.docx.

## Data Availability

The datasets generated and analyzed during the current study are available in the National Biotechnology Information Center (NCBI) GenBank database with the accession number CP174089.
