# Supplementary figures and images for: Phenotypic and Genetic Characterization and Production Abilities of Lacticaseibacillus rhamnosus Strain 484—A New Probiotic Strain Isolated From Human Breast Milk
Source: Food Sci Nutr. 2025 Sep 26;13(10):e70980. doi: 10.1002/fsn3.70980 (PMC12464452; doi:10.1002/fsn3.70980)

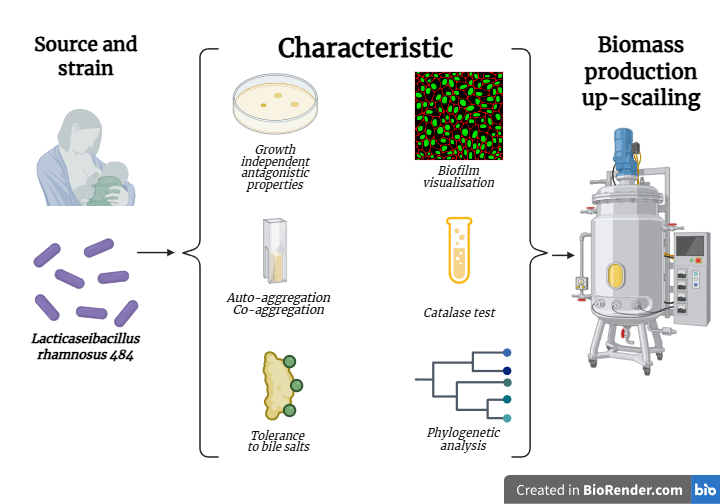

Supplement: Supplementary file 1 — Data S1: fsn370980‐sup‐0001‐Supinfo.png. [file FSN3-13-e70980-s002.png]
