# Supplementary material for: Phenotypic and Genetic Characterization and Production Abilities of Lacticaseibacillus rhamnosus Strain 484—A New Probiotic Strain Isolated From Human Breast Milk
Source: Food Sci Nutr. 2025 Sep 26;13(10):e70980. doi: 10.1002/fsn3.70980 (PMC12464452; doi:10.1002/fsn3.70980)
Supplement: Supplementary file 2 — Data S2: fsn370980‐sup‐0002‐TableS1.docx. [file FSN3-13-e70980-s001.docx]

Supplementary Table 1. List of MGE elements detected in *L. rhamnosus* 484 genome by MobileElementFinder tool.

| **No** | **Name** | **Synonyms** | **Prediction** | **Type** | **Lenght** | **Identity** | **Substitution** |
| --- | --- | --- | --- | --- | --- | --- | --- |
| 1 | ISLrh4 |  | alignment to reference | Insertion sequence | 1577 | 1 | 0 |
| 2 | ISLrh4 |  | alignment to reference | Insertion sequence | 1577 | 1 | 0 |
| 3 | ISLrh4 |  | alignment to reference | Insertion sequence | 1577 | 1 | 0 |
| 4 | ISLrh4 |  | alignment to reference | Insertion sequence | 1577 | 1 | 0 |
| 5 | ISLrh4 |  | alignment to reference | Insertion sequence | 1577 | 1 | 0 |
| 6 | ISLrh2 | IS11 | alignment to reference | Insertion sequence | 1575 | 0,99 | 14 |
| 7 | ISLrh2 | IS11 | alignment to reference | Insertion sequence | 1575 | 0,99 | 14 |
| 8 | ISLrh2 | IS11 | alignment to reference | Insertion sequence | 1575 | 0,99 | 14 |
| 9 | ISLrh2 | IS11 | alignment to reference | Insertion sequence | 1575 | 0,99 | 14 |
| 10 | ISLrh2 | IS11 | alignment to reference | Insertion sequence | 1575 | 0,99 | 14 |
| 11 | ISLrh2 | IS11 | alignment to reference | Insertion sequence | 1575 | 0,99 | 14 |
| 12 | ISLrh2 | IS11 | alignment to reference | Insertion sequence | 1575 | 0,99 | 14 |
| 13 | ISLrh2 | IS11 | alignment to reference | Insertion sequence | 1575 | 0,99 | 14 |
| 14 | ISLrh2 | IS11 | alignment to reference | Insertion sequence | 1575 | 0,99 | 14 |
| 15 | ISLrh2 | IS11 | alignment to reference | Insertion sequence | 1575 | 0,99 | 14 |
| 16 | ISLrh2 | IS11 | alignment to reference | Insertion sequence | 1575 | 0,99 | 14 |
| 17 | ISLrh2 | IS11 | alignment to reference | Insertion sequence | 1575 | 0,99 | 14 |
| 18 | ISLrh2 | IS11 | alignment to reference | Insertion sequence | 1575 | 0,99 | 14 |
| 19 | ISLrh2 | IS11 | alignment to reference | Insertion sequence | 1575 | 0,99 | 14 |
| 20 | ISLrh2 | IS11 | alignment to reference | Insertion sequence | 1575 | 0,99 | 14 |
| 21 | ISLrh2 | IS11 | alignment to reference | Insertion sequence | 1575 | 0,99 | 13 |
| 22 | ISLrh2 | IS11 | alignment to reference | Insertion sequence | 1575 | 1,00 | 2 |
| 23 | ISLrh2 | IS11 | alignment to reference | Insertion sequence | 1575 | 1,00 | 2 |
| 24 | ISLrh2 | IS11 | alignment to reference | Insertion sequence | 1575 | 1,00 | 2 |
| 25 | ISLrh2 | IS11 | alignment to reference | Insertion sequence | 1575 | 1,00 | 2 |
| 26 | ISLrh2 | IS11 | alignment to reference | Insertion sequence | 1575 | 1,00 | 2 |
| 27 | ISLrh2 | IS11 | alignment to reference | Insertion sequence | 1575 | 1,00 | 2 |
| 28 | ISLrh3 |  | alignment to reference | Insertion sequence | 1564 | 1,00 | 0 |
| 29 | ISLrh3 |  | alignment to reference | Insertion sequence | 1564 | 1,00 | 0 |
| 30 | cn_16187_ISLrh4 | | inferred | Composite transposon | 16187 | 1,00 | 0 |
| 31 | cn_36734_ISLrh2 | | inferred | Composite transposon | 36734 | 0,99 | 14 |
| 32 | cn_10002_ISLrh2 | | inferred | Composite transposon | 10002 | 0,99 | 14 |
| 33 | cn_18672_ISLrh2 | | inferred | Composite transposon | 18672 | 0,99 | 13 |
| 34 | cn_49798_ISLrh2 | | inferred | Composite transposon | 49798 | 0,99 | 14 |
| 35 | cn_5326_ISLrh2 | | inferred | Composite transposon | 5326 | 0,99 | 14 |
| 36 | cn_41817_ISLrh2 | | inferred | Composite transposon | 41817 | 0,99 | 14 |
| 37 | cn_8887_ISLrh2 | | inferred | Composite transposon | 8887 | 0,99 | 14 |
| 38 | cn_42697_ISLrh2 | | inferred | Composite transposon | 42697 | 0,99 | 14 |
| 39 | cn_35666_ISLrh2 | | inferred | Composite transposon | 35666 | 1,00 | 2 |
| 40 | cn_11109_ISLrh2 | | inferred | Composite transposon | 11109 | 0,99 | 14 |
